# Popularity and customer preferences for over-the-counter Chinese medicines perceived by community pharmacists in Shanghai and Guangzhou: a questionnaire survey study

**DOI:** 10.1186/1749-8546-9-22

**Published:** 2014-09-13

**Authors:** Shuai Ge, Tian-Tian He, Hao Hu

**Affiliations:** 1 State Key Laboratory of Quality Research in Chinese Medicine, Institute of Chinese Medical Sciences, University of Macau, Macau, China

## Abstract

**Background:**

This study interviewed community pharmacists in Shanghai and Guangzhou for their perception of the popular categories of over-the-counter (OTC) Chinese medicines and the factors affecting customer preferences for OTC Chinese medicines.

**Methods:**

A cross-sectional survey was carried out in six main administrative districts in Guangzhou and eight main administrative districts in Shanghai, China. Descriptive statistical analysis was conducted in this study.

**Results:**

OTC Chinese medicines contributed 21–50% among all the pharmaceutical sales by the community pharmacies. The prevalent categories of OTC Chinese medicines were common cold medicines, respiratory system medicines, digestive system agents, gynecological medicines, health tonic medicines, and *qing re (heat-clearing*) and *qu du* (*detoxifying*) medicines. Customers were more concerned about medical factors of OTC Chinese medicines than business factors. Among the medical factors, the most important was drug safety, followed by efficacy, contraindications, indications, and side effects. Among the business factors, the most important were brand and price.

**Conclusions:**

This study identified the top sales categories of OTC Chinese medicines in Shanghai and Guangzhou and the important factors such as drug safety, efficacy, period of validity, contraindications, and indications that are affecting the customer preferences for OTC Chinese medicines.

## Background

Self-medication is becoming an increasingly important part of global healthcare systems where aging populations experience a higher prevalence of chronic medical conditions [1,2], including China where the pace of aging has been accelerating in the past decade [3]. Convenient self-medication with fewer adverse drug reactions is preferred [4]. Consequently, over-the-counter (OTC) medicines are gaining a significant presence in the pharmaceutical sector. For example, US sales of OTC Chinese medicines were about 1.7 billion US dollar in 2010 and there are approximately 100 000 OTC products available in the US market [5,6]. These demographic and socioeconomic changes present opportunities for Chinese medicines in the OTC market.

Chinese medicines confer advantages in the self-medication OTC market and increase drug accessibility for patients [7]. Specifically, OTC Chinese medicines are usually used without professional supervision [8], and their target mild diseases and chronic medical conditions do not need regular dose adjustments for long periods of time [9-11]. In addition, OTC Chinese medicines are more widely accepted than conventional Western medicines (WM) because of Chinese patients’ beliefs and preferences for natural products and alternative medicines [12-14].

The factors that affect customer preferences for OTC Chinese medicines are under study to promote research and development (R&D) of OTC Chinese medicines. As an important part of research on traditional Chinese medicine (TCM), R&D of OTC Chinese medicines will contribute to the proposal of priority areas for future research and new drug innovations. Yu [15] proposed that medicinal products’ quality and brand are the most critical factors for OTC Chinese medicine acceptance by customers. Toxic contaminants in OTC Chinese medicines are frequently discussed as a safety issue [16,17]. The appropriate dosages and schedules required to avoid common adverse reactions have also been the subject of research [18,19]. Moreover, researchers have begun to pay more attention to the cognitive preferences for OTC Chinese medicines [20]. Hon *et al.*[21] tested pharmacy students’ attitudes toward OTC Chinese medicines, and discovered that pharmacy students’ cognitive understanding of Chinese medicines significantly affected their attitudes toward OTC Chinese medicines. Chung *et al.*[22] investigated the use of OTC and prescription medicines across WM and TCM among urban Chinese patients and found that suggestions from healthcare professionals were highly relevant to patients’ preferences for use of OTC products, regardless of whether WM or TCM was involved. However, related empirical studies remained limited and did not provide adequate knowledge of customer preferences for the development of OTC Chinese medicines.

Hospitals and community pharmacies were two main distribution channels for OTC Chinese medicines in China. Chinese medicines were widely used for treating cardiovascular and cerebrovascular diseases in hospitals (Additional file 1). This was followed by their use for cancer, and Chinese medicines were reported to cause less suffering and have significant efficacy in cancer treatment [23]. Chinese medicines were also widely used to treat respiratory diseases, musculoskeletal diseases, gynecological diseases, and urinary system diseases in hospitals. However, the most prevalent Chinese medicines used in hospitals were mostly Chinese medicine injections and belonged to the prescription medications [24]. OTC Chinese medicines did not have a significant presence in hospitals compared with prescription Chinese medicines [25,26].

Consequently, the most important market for OTC Chinese medicines in China is the community pharmacy [27,28]. However, no previous publications have focused on the popularity of OTC Chinese medicines in community pharmacies. The popular categories of OTC Chinese medicines in the community pharmacy market remained undefined and the factors affecting customer preferences for OTC Chinese medicines in community pharmacies were unknown.

This study aims to survey the popular categories of OTC Chinese medicines in community pharmacies in China and the factors affecting customer preferences for OTC Chinese medicines.

## Methods

### Data collection

This study used a paper-form questionnaire to collect first-hand data about OTC Chinese medicines in community pharmacies. The survey design was reviewed and approved by the Ethics Committee of the Research Committee at the University of Macau. Shanghai and Guangzhou were chosen as the target cities for this study because of their economic backgrounds, huge populations of TCM users, and citizens’ acceptance toward TCM products as OTC Chinese medicines [29]. In more detail, the sales within the TCM industry in Guangdong province were 8.9 billion RBM, and accounted for 9.1% of the whole country sales in 2005. The proportion of facilities with a TCM department among all 228 community health centers in Shanghai was 93.4% in 2006. The two cities also have centralized leading TCM science and research institutes, including over 30 TCM research laboratories in Shanghai and 21 research institutes in Guangzhou [30-32]. The TCM culture in the two cities is also time-honored, for instance, there are more than four old-brand TCM enterprises established before the 19th century, indicating a long history of TCM use in Guangzhou [33].

Therefore, the information collected from community pharmacies in both cities could provide informative results about the popularity and customer preferences for OTC Chinese medicines in China.

The naive theory of popularity explained the belief that a product is desirable when it is popular [34,35]. The concept “popularity” was defined as a mixture of best-selling and prevalence, with the aim of indicating the market potential of OTC products. Because there was no reliable statistical information about the total number of community pharmacies in both cities, stratified sampling from different city districts was conducted to ensure that the study could collect information from community pharmacies located in different social and economic environments. All of the main administrative districts in Guangzhou were chosen, namely Tianhe, Yuexiu, Baiyun, Liwan, Huangpu, and Dongshan. The survey also covered all of the main administrative districts in Shanghai, including Huangpu, Luwan, Xuhui, Jingan, Putuo, Yangpu, Minhang, and Jinshan. The whole survey was conducted and completed in May 2013.

The pharmacist in charge of pharmaceutical services at each community pharmacy was targeted as the survey respondent. During visits to the community pharmacies, one researcher involved in the study as an investigator obtained informed consent in oral form from the respondents before the formal survey, following a standardized process: (1) introducing the researcher herself at first; (2) explaining research background and research objective in a structural way, with the cover page of questionnaire (Additional file 2); (3) clarifying the academic and anonymous nature of this survey; and (4) consulting the agreement of respondents. If there were any doubts in the survey, the investigator would spell out more specific information for the respondents in a word-by-word manner. The questionnaires were completed by the community pharmacists themselves without any intervention. All of the questionnaires were distributed directly to the respondents and collected on site in the same visit.

### Measurements

A semi-structured questionnaire (Additional file 2) was used in this study. The questionnaire was composed of three parts. The first part solicited background information about the community pharmacy, specifically the year of establishment, number of employees, business area, geographic position, form of ownership, management model (chain drugstore or not), involvement in social medical security (yes or no), average number of daily customers, and provision of medical care services (yes or no).

The second part of the questionnaire solicited information about the popularity of OTC Chinese medicines. The respondents were asked to choose the sales proportions of four items: pharmaceutical sales/gross turnover; prescription medicine sales/pharmaceutical sales; OTC medicine sales/pharmaceutical sales; and OTC Chinese medicine sales/pharmaceutical sales. In addition, the respondents were asked to write down the five most popular categories of OTC Chinese medicines and the three best-selling products in each category.

The third part of the questionnaire investigated the factors affecting customer preferences for OTC Chinese medicines. The possible factors were divided into medical factors and business factors. The medical factors of OTC Chinese medicines referred to medicine safety, efficacy, dosage form, indications, contraindications, period of validity, and side effects. The business factors of OTC Chinese medicines referred to price, brand, production area, packaging, and label design, and whether the medicine was listed in the Catalogue of Drugs for Basic National Medical Insurance and Countermeasures of China (the Catalogue) [36]. The respondents were asked about the extent to which, in their experience, each factor influenced customer preferences for OTC Chinese medicines, measured on a 5-point Likert scale (1 = not at all; 5 = very great). After the choices made by each respondent, the degree of influence was defined as the total choices related to each level (not at all, very great, or others) divided by the total number of respondents in percentages.

### Data analysis

Preliminary analyses of the data collected in this study showed that there were no significant differences among the cities, geographic positions, forms of ownership, and business areas of the community pharmacies. Overall, the study aimed to provide a comprehensive exploration of OTC Chinese medicines in the community pharmacy market, rather than specific tests of community pharmacy factors. Therefore, the survey questionnaire data were analyzed through descriptive statistics. All the data for the background information of the community pharmacies, sales proportion items, and factors affecting customer preferences were analyzed through percentage distributions. The data for the popular categories of OTC Chinese medicines were analyzed by ordination methods through counting frequencies. The data for the popular products in each category were presented in word descriptions.

## Results

### Background information for the community pharmacies evaluated

A total of 108 community pharmacies in Guangzhou were visited, and 51 respondents completed the questionnaire. In Shanghai, 137 community pharmacies were visited, and 52 respondents completed the questionnaire. In total, 103 completed questionnaires were collected, with a response rate of 42%, reflecting the difficulties in acquiring the cooperation of community pharmacists [37]. After deleting questionnaires with missing answers, 100 valid questionnaires were finalized for further analyses.

The year of establishment for the community pharmacies evaluated ranged from 1992 to 2012. On average, the community pharmacies had operated for 8 years. The number of employees ranged from 2 to 63, with an average of 9.82 per community pharmacy.

As shown in Table 1, 42% of the community pharmacies had a business area of 50–100 m^2^ and 38% of the community pharmacies had a business area of 100–150 m^2^. The majorities (78%) of the community pharmacies evaluated were located in a residential community, 20% were in a business community, and the final 2% were in remote areas. The majority (86%) of the community pharmacies were privately owned, 6% were state-owned, 4% belonged to foreign investors, and 4% were joint ventures between two ownership groups.

**Table 1:** Background information of the studied community pharmacies (N = 100)

**Background**		**Percentage**
Geographic position	Residential community	78%
	Business community	20%
	Remote areas	2%
Form of ownership	Private	86%
	State-owned	6%
	Foreign-owned	4%
	Joint venture	4%
Management model	Chain drugstore	64%
	Single drugstore	36%
Involved in social medical security	Yes	52%
	No	48%
Offer medical care services	Yes	20%
	No	80%
Business areas	0-50 m^2^	2%
	51-100 m^2^	42%
	101-150 m^2^	38%
	151-200 m^2^	6%
	> 200 m^2^	12%
Average number of drug-purchasing customers daily	0-50	14%
	51-100	30%
	101-150	16%
	151-200	16%
	201-250	6%
	> 250	18%

Sixty-four per cent of the community pharmacies were chain drugstores and 36% were single drugstores. Fifty-two per cent of the community pharmacies were involved in China’s social medical security system. Through this system, Chinese citizens can defray some of the costs of medical expenses in the event of illness or injury, including expenditure on OTC Chinese medicines. However, only 20% of the community pharmacies offered medical care services. Thirty per cent of the community pharmacies served an average of 51–100 drug-purchasing customers daily and 18% of the community pharmacies served over 250 drug-purchasing customers each day.

All community pharmacies sold both conventional medicines and Chinese medicines. OTC medicines contributed more toward sales income than prescription medicines. OTC Chinese medicines contributed 21–50% of the income from pharmaceutical sales for the majority of the community pharmacies (Additional file 3).

### Popular categories of OTC Chinese medicines in the community pharmacies

The six most popular categories of OTC Chinese medicines were cold medicines, respiratory system medicines, digestive system agents, gynecological medicines, health tonic medicines, and heat-clearing and detoxifying medicines (Table 2). The total frequency of these six categories in the data was approximately 81%.

**Table 2:** Categories of OTC Chinese medicine in the community pharmacies

**Category no.**	**Categories**	**Frequency**	**Percentage**
1	Cold medicine	91	18%
2	Respiratory system medicine	72	14%
3	Digestive system agent	67	13%
4	Gynaecological medicine	63	13%
5	Health tonic medicine	60	12%
6	*Heat-clearing and *detoxifying* medicine	54	11%
7	ENT medicine	28	6%
8	Dermatology medicine	24	5%
9	Circulatory system agent	17	3%
10	Orthopaedics medicine	15	3%
11	Summer humidity agent	8	2%
12	Nervous system medicine	1	0%
Total	—	500	100%

*Heat-clearing and *detoxifying* means *Qingre Jiedu*.

According to respondents’ answers about the sales situation for OTC Chinese medicine products, the most popular products in each of the six most prevalent categories of OTC Chinese medicines were summarized in ordination (Table 3). These items comprised common products in OTC markets. For example, Xiaochaihu granules, made with the traditional Chinese medicines *Bupleurum chinense* and *Scutellaria baicalensis*, have obvious curative powers for flu and gastrointestinal dysfunction [38].

**Table 3:** Popular products within the six most popular categories

**Categories**	**Popular products**
Cold medicine	999 Ganmaoling granule
	Xiaochaihu granule
	Vitamin C Yinqiao tablet
	Antiviral oral-liquid
	Lianhua Qingwen capsule
Respiratory system medicine	Milian Chuanbei Pipa paste
	999 Qiangli Pipa lu
	Shedan Chuanbei liquid
	Chuanbei Pipa paste
	Juhong tanke liquid
Digestive system agent	Huoxiang Zhengqi liquid (pill, capsule, liquid)
	Jianwei Xiaoshi tablets
	999 Weitai granules
	Stomach-recovering capsule
	Health pill
Gynaecological medicine	Fuyankang tablets
	Gynecologic Qianjin tablets
	Wuji Baifen Wan
	Huahong tablets
	Kanggongyan tablets
Health tonic medicine	Donkey-hide gelatin (syrup, piece, particle, liquid)
	Liuwei Dihuang pill
	Renal Aid
	Buzhong Yiqi pill
	Anshen Bunao syrup
*Heat-clearing* and *detoxifying* medicine	Banlangen granules
	Niuhuang Jiedu tablets
	Zhongsheng pill
	Sanhuang tablets
	Pudilan Xiaoyan tablets

### Factors affecting customer preferences for OTC Chinese medicines

**Figure 1:**
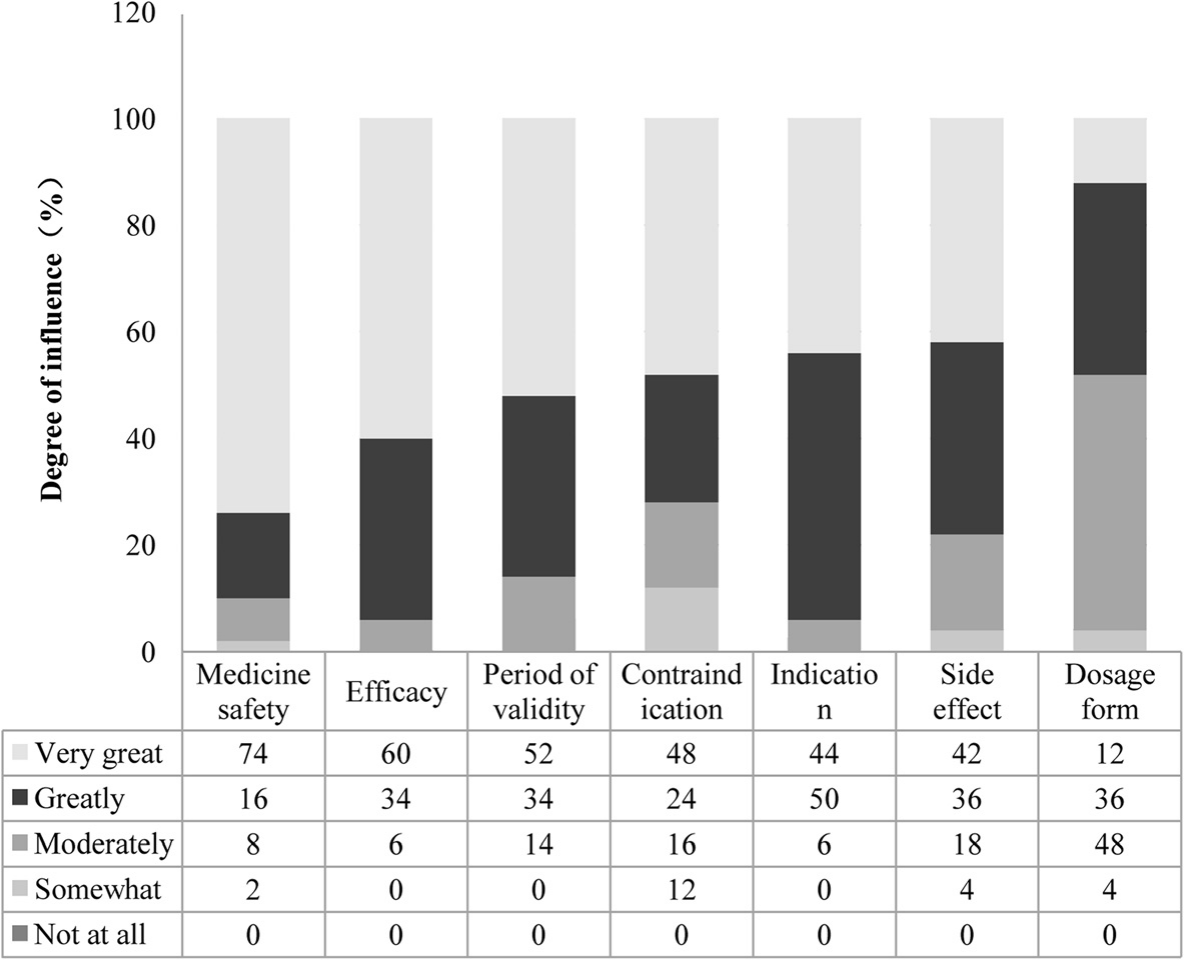
Medical factors affecting customer preference for OTC Chinese medicine.

The medical factors were the key factors affecting customer preferences for OTC Chinese medicines. Of the medical factors, medicine safety was the most important. The next most important factors were efficacy and period of validity. The factors of contraindications, indications, and side effects also had strong effects on customer decision-making. The dosage form did not have a strong effect on customer preferences (Figure 1).

**Figure 2:**
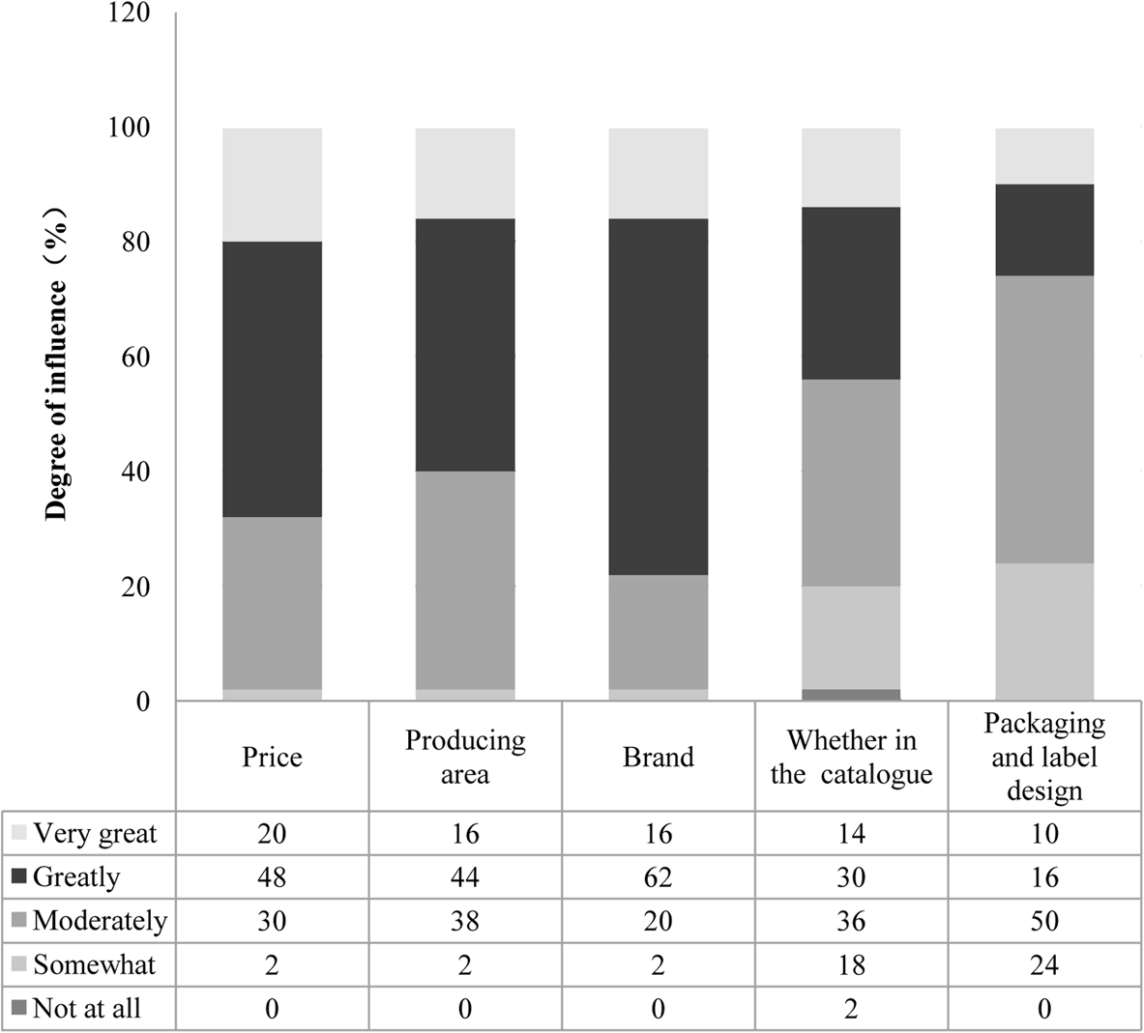
Business factors affecting customer preference for OTC Chinese medicine.

The business factors, namely price, production area, brand, inclusion in the Catalogue, packaging, and label design, did not have such clear effects on customer preferences for OTC Chinese medicines. The medicine brand was a relatively more important factor, as were price and production area. Patients rarely considered packaging and label design, and did not consider whether their OTC Chinese medicine purchases were listed in the Catalogue, implying that their decision-making was not influenced by whether they would be reimbursed for their OTC Chinese medicines (Figure 2). As mentioned before, China’s social medical insurance system was established to provide medical reimbursement and basic medical services to citizens, and the Catalogue is an important part of the system [39]. The Catalogue is able to balance the prices in the OTC market to some degree by adjusting the OTC medicines listed in the Catalogue [40]. However, similar to the consumer awareness of drug prices, the attention paid to the Catalogue was relatively low.

## Discussion

Based on the survey data, OTC Chinese medicines accounted for a high proportion of pharmaceutical sales in the community pharmacies evaluated, suggesting that OTC Chinese medicines have wide acceptance by Chinese customers and great potential in the self-medication market in China, especially through the distribution channel of community pharmacies.

The most common uses of Chinese medicines in hospitals are for the treatment of cardiovascular diseases, cancer, and other serious illnesses (see Additional file 1), which are obviously different from the uses of OTC Chinese medicine at community pharmacy. Thus, OTC Chinese medicine producers could make use of their comparative advantages for common diseases and health maintenance areas, which have been regarded as one of the main strengths of Chinese medicines [41]. Thus, future R&D by OTC Chinese medicine producers could focus on the common disease areas targeted by Chinese medicines. For instance, Valerian extract capsules for the treatment of digestive system diseases and Jinggan capsules for the treatment of common colds were developed in 2011, and both focus on common disease areas [42]. Future R&D could also focus on the further development of existing OTC Chinese medicines and the improvement of their dose calculations to enlarge their market existence and increase customer acceptance.

According to the perceptions of community pharmacists, medicine safety and other medical factors were the key factors affecting customer preferences for OTC Chinese medicines. The community pharmacists surveyed thought that their customers were influenced more by medical factors than by business factors. Therefore, medical factors, comprising medicine safety, efficacy, period of validity, contraindications, indications, and side effects, were more influential factors for customers when choosing OTC Chinese medicines. Although most Chinese medicines are developed from natural products, safety problems have frequently been reported [43,44]. Over the past decade, a number of OTC Chinese medicines have been featured in the news after patients developed serious adverse effects arising from problems with the products, such as high heavy metal levels found in an aloe compound capsule and acute kidney injury caused by Han fang ji (*Stephania tetrandra*) [45]. Another medicine safety issue is improper use in self-medicating because of lack of knowledge [46-48]. Some OTC Chinese medicines might be subject to inadequate quality control and contain harmful additives or unlisted ingredients [49,50]. Furthermore, interactions between different products used simultaneously might cause serious problems [51,52].

Therefore, medicine safety should be the most important factor under consideration in drug use, especially the pharmacological, pharmacodynamics, and toxicological aspects, and adverse reactions of OTC Chinese medicines [53], as also identified in this study. In particular, rigorous guidelines and clinical trials specific to OTC Chinese medicines were required to be advanced and applied to ensure that clinical research is honest and reliable [54]. In addition, unsafe OTC Chinese medicines might arise from unprofessional practices by manufacturers [55]. Some of these problems could be controlled by the development of standard operating procedures for the industry, including good agricultural practice, good supply practice, and good manufacturing practice for implementation during OTC Chinese medicine production [56].

Some factors did not significantly affect the customer preferences for OTC Chinese medicines, such as dosage form, packaging, and label design. Reimbursement and price were also not important. This might be because the medical security system in China was improved after the New Healthcare Reform, which included the addition of many more OTC Chinese medicines to the Catalogue [57]. Overall, 203 Chinese medicines were listed in the national essential drugs lists in 2012, occupying approximately one-third of the whole Catalogue [58]. Moreover, OTC Chinese medicines always have a competitive price advantage over most conventional WM [59,60]. Thus, OTC Chinese medicines have many opportunities for growth in therapeutic areas, and it will be rewarding for producers to invest more money into R&D for OTC Chinese medicines.

Several study limitations should be addressed in future studies. First, the survey used for data collection was restricted to specific areas. Although Shanghai and Guangzhou could provide information about the main consumption of OTC Chinese medicines in China, the results may not accurately represent the situations in middle- and small-sized cities. Future studies covering more geographical areas would provide more complete information about OTC Chinese medicines in different economic and social contexts. Second, this study was based on the perceptions of community pharmacists rather than direct measurements of OTC Chinese medicine consumption. Future studies could be advanced through the creation of new methods to collect direct consumption data. Third, this study used an overview of the popular categories and products of OTC Chinese medicines. Further research could focus on specific categories. The treatment areas of cold medicines and digestive system agents, for example, contain various complex OTC Chinese medicines and require more detailed study.

## Conclusions

This study identified the top sales categories of OTC Chinese medicines in Shanghai and Guangzhou and the important factors such as drug safety, efficacy, period of validity, contraindications, and indications that are affecting the customer preferences for OTC Chinese medicines.

## Competing interests

The authors declare that they have no competing interests.

## Authors’ contributions

SG and HH conceived and designed the study. SG conducted the fieldwork. SG, TTH and HH performed the data analysis. TTH and HH wrote the manuscript. All authors read and approved the final manuscript.

## Supplementary Material

Additional file 1 Shares of Chinese medicines in hospital (2005-2011). Click here for file

Additional file 2 Popularity and customer preference of OTC Chinese medicine questionnaire. Click here for file

Additional file 3 Sales proportion in community pharmacies. Click here for file
