# Supplementary material for: Popularity and customer preferences for over-the-counter Chinese medicines perceived by community pharmacists in Shanghai and Guangzhou: a questionnaire survey study
Source: Chin Med. 2014 Sep 13;9:22. doi: 10.1186/1749-8546-9-22 (PMC4169131; doi:10.1186/1749-8546-9-22)
Supplement: Additional file 1 — Shares of Chinese medicines in hospital (2005-2011). [file 1749-8546-9-22-S1.docx]

**Supplementary file 1: Shares of Chinese medicines in hospital (2005-2011)**

| **Categories** | **2005** | **2006** | **2007** | **2008** | **2009** | **2010** | **2011** |
| --- | --- | --- | --- | --- | --- | --- | --- |
| C[ardiovascular](app:ds:cardiovascular) [and](app:ds:and) [cerebrovascular](app:ds:cerebrovascular) [diseases](app:ds:diseases) | 38.86% | 36.68% | 37.34% | 37.42% | 37.40% | 37.71% | 37.12% |
| Cancer | 15.63% | 14.87% | 15.41% | 15.94% | 16.02% | 15.93% | 15.23% |
| Respiratory system disease | 12.31% | 12.87% | 11.36% | 11.15% | 11.51% | 10.95% | 10.88% |
| Musculoskeletal disease | 5.79% | 8.31% | 8.11% | 8.29% | 7.92% | 8.28% | 9.19% |
| [Digestive](app:ds:digestive) [system](app:ds:system) [disease](app:ds:disease) | 8.67% | 7.30% | 6.97% | 6.47% | 6.31% | 6.19% | 6.17% |
| [Gynaecological disease](app:ds:gynecological%20disease) | 4.78% | 4.87% | 5.16% | 5.49% | 5.39% | 5.30% | 5.21% |
| Urinary system disease | 3.72% | 4.29% | 4.16% | 4.28% | 4.30% | 4.41% | 4.90% |
| Nerve system disease | 2.87% | 2.95% | 3.53% | 3.26% | 3.41% | 3.48% | 3.70% |
| Ophthalmology and otorhinolaryngology | 3.03% | 2.89% | 2.76% | 2.47% | 2.42% | 2.52% | 2.62% |
| [Dermatology](app:ds:dermatology) | 0.95% | 1.61% | 1.68% | 1.72% | 1.80% | 1.91% | 1.86% |
| Supplementing and boosting Chinese medicines | 1.64% | 1.36% | 1.27% | 1.25% | 1.26% | 1.31% | 1.35% |
| Paediatric | 0.93% | 0.87% | 0.86% | 0.82% | 1.00% | 1.06% | 0.91% |
| Others | 0.81% | 1.13% | 1.41% | 1.44% | 1.27% | 0.95% | 0.88% |
| Total | 100% | 100% | 100% | 100% | 100% | 100% | 100% |

Source: China Food and Drug Administration’s Southern Medicine Economic Research Institute: China Medicine Economic Information Network (<http://www.menet.com.cn>)
