# Supplementary material for: Popularity and customer preferences for over-the-counter Chinese medicines perceived by community pharmacists in Shanghai and Guangzhou: a questionnaire survey study
Source: Chin Med. 2014 Sep 13;9:22. doi: 10.1186/1749-8546-9-22 (PMC4169131; doi:10.1186/1749-8546-9-22)
Supplement: Additional file 2 — Popularity and customer preference of OTC Chinese medicine questionnaire. [file 1749-8546-9-22-S2.docx]

**Supplementary file 2: Popularity and customer preference of OTC Chinese medicine questionnaire**

To the managers of community pharmacies:

This questionnaire is only for understanding of the sales status of over-the-counter (OTC) Chinese medicines in community pharmacies, including the sales turnovers, popular OTC categories et al., in order to make reasonable suggestions for OTC Chinese medicine development. This questionnaire is absolutely anonymous; definitely it will not be involved with any commercial interests. Thanks for your participation!

(Please make “√” or write down in “____” with your answers. Make a single choice unless otherwise noted.)

**Part 1 background information**

1．The establishment year of community pharmacy（____）year

2．Number of employees of community pharmacy （____）person

3．Business area of community pharmacy.

□0-50 m^2^ □50-100 m^2^ □100-150 m^2^ □150-200 m^2^ □above 200 m^2^

4．Geographic position of community pharmacy.

□Business community □Residential community □Remote areas

5．Form of ownership of community pharmacy.

□Privately-owned □State-owned □Foreign-invested □Joint ventures

6．Management model of community pharmacy.

□Chain drugstore □Single drugstore

7．Involvement in social medical security of community pharmacy.

□Yes □No

8．Average daily purchasers of community pharmacy.

□0-50 □51-100 □101-150 □151-200 □201-250 □above250

9．Provision of medical care services of community pharmacy.

□Yes □No

**Part 2 Popularity status of OTC Chinese medicine**

1．Drug sales percentage of total turnover.

□0-10% □10-20% □20-30% □30-40% □40-50%

□50-60% □60-70% □70-80% □80-90% □90-100%

2．Prescription drug sales percentage of total drug turnover.

□0-10% □10-20% □20-30% □30-40% □40-50%

□50-60% □60-70% □70-80% □80-90% □90-100%

3．OTC drug sales percentage of total drug turnover.

□0-10% □10-20% □20-30% □30-40% □40-50%

□50-60% □60-70% □70-80% □80-90% □90-100%

4．Chinese medicine sales percentage of total drug turnover.

□0-10% □10-20% □20-30% □30-40% □40-50%

□50-60% □60-70% □70-80% □80-90% □90-100%

5．Popular categories of OTC Chinese medicine：

(Please sort the following categories of OTC Chinese medicine by sales from top to bottom, and fill the three products with the highest sales in top five categories in the blanks.)

A. Orthopaedics medicine B. Circulatory system agent C. Health tonic medicine D. Respiratory system medicine E. ENT medicine F. Gynaecological medicine G. Cold medicine H. Digestive system agent. I. Heat-clearing and detoxifying medicine J. Nervous system medicine K. [Dermatology](app:ds:dermatology) medicine L. Summer humidity agent

(For instance：(G)：①Xiaochaihu granules②999 Ganmaoling granules③Vitamin C Yinqiao tablets (for reference only))

Please fill in the actual situation：

1.（____）: ①____②____③____

2.（____）: ①____②____③____

3.（____）: ①____②____③____

4.（____）: ①____②____③____

5.（____）: ①____②____③____

**Part 3 Factors affecting consumer preferences of OTC Chinese medicine**

To what extent, in your experience, each factor influences customer preference of OTC Chinese medicine, measuring on a 5-point Likert scale (1 = not at all; 5 = very great).

***Business nature factors:***

1．Medicine price：

A. Not at all B. Somewhat C. Moderately D. Greatly E. Very great

2．Medicine brand：

A. Not at all B. Somewhat C. Moderately D. Greatly E. Very great

3．Medicine producing area：

A. Not at all B. Somewhat C. Moderately D. Greatly E. Very great

4．Packaging and label design of medicines：

A. Not at all B. Somewhat C. Moderately D. Greatly E. Very great

5．Whether the medicine is listed in the Catalogue of Drugs for Basic National Medical Insurance and Countermeasures of China or not：

A. Not at all B. Somewhat C. Moderately D. Greatly E. Very great

***Medical nature factors:***

6．Medicine safety：

A. Not at all B. Somewhat C. Moderately D. Greatly E. Very great

7．Medicine efficacy：

A. Not at all B. Somewhat C. Moderately D. Greatly E. Very great

8．Dosage forms of medicines：

A. Not at all B. Somewhat C. Moderately D. Greatly E. Very great

9．Adaptation disease of medicines：

A. Not at all B. Somewhat C. Moderately D. Greatly E. Very great

10．Medicine period of validity：

A. Not at all B. Somewhat C. Moderately D. Greatly E. Very great

11．Medicine side effects：

A. Not at all B. Somewhat C. Moderately D. Greatly E. Very great

12．Medicine contraindication：

A. Not at all B. Somewhat C. Moderately D. Greatly E. Very great

零售藥店中成藥銷售情況調查問卷

各位社區藥店負責人：

你們好！本調查的目的是瞭解目前中成藥在零售藥店的銷售情況，包括中成藥銷售概況、主要銷售品種及其影響因素等，以期對中成藥零售市場的發展提出合理建議。

本研究純屬學術研究，本調查採取匿名形式！不涉及任何商業利益！

請您就一下問題在您認為合適的地方打“√”或在“____”處填上適當的內容。（注：除含特殊標注的題目外，其餘均為單項選擇題）

感謝您的配合及協助！

澳門大學 中華醫藥研究院

第一部份 藥店基本信息

1．藥店成立年份？（____）年

2．藥店的員工數？（____）人

3．藥店營業面積？

□0-50m2□50-100m2□100-150m2□150-200m2□200m2以上

4．藥店的地理位置？

□商業區附近 □居民區（社區）附近 □其他偏遠地段

5．藥店的所有制形式？

□民營（私人/個體） □國有 □外資 □其它

6．藥店的經營性質？

□連鎖藥店□單體藥店

7．藥店是否被納入社保醫療定點藥店？

□是□否

8．藥店平均每日購藥人次？

□0-50人□50-100人□100-150人□150-200人□200-250人□250人以上

9．藥店是否提供醫生診療服務？（如：坐堂醫等）

□是□否

第二部分中成藥銷售概況

1．藥品銷售收入占總營業額的百分比？

□0-10%□10-20%□20-30%□30-40% □40-50%

□50-60%□60-70%□70-80% □80-90%□90-100%

2．處方藥銷售收入占藥品總營業額的百分比？

□0-10%□10-20%□20-30%□30-40% □40-50%

□50-60%□60-70%□70-80% □80-90%□90-100%

3．非處方藥銷售收入占藥品總營業額的百分比？

□0-10%□10-20%□20-30%□30-40% □40-50%

□50-60%□60-70%□70-80% □80-90%□90-100%

4．中成藥銷售收入占藥品總營業額的百分比？

□0-10%□10-20%□20-30%□30-40% □40-50%

□50-60%□60-70%□70-80% □80-90%□90-100%

5．藥店銷售中成藥的主要類別：（請將下列類別按照銷售額進行排序並將前五位的序號填入下面的括弧內，並請在每個類別後面的“”上列出該類別銷售額前三位元的中成藥產品）

A.骨傷科用藥B.循環系統用藥C.補益養生類藥D.呼吸系統用藥

E.五官科用藥F.婦科用藥G.感冒用藥H.消化系統用藥

I.清熱解毒藥J.神經系統用藥K.皮膚科用藥L.暑濕類

（舉例：（G）：①小柴胡顆粒②999感冒靈顆粒③維C銀翹片（隨意列舉，僅供參考））

請根據實際情況進行填寫：

1、（）：①____②____③____

2、（）：①____②____③____

3、（）：①____②____③____

4、（）：①____②____③____

5、（）：①____②____③____

第三部分消費者選購中成藥的關注因素

依據您的經驗，消費者在您的藥店選購中成藥時，在多大程度上關注下列因素：

商品屬性：

1．藥品價格：

A.根本不關注B.不太關注C.一般D.比較關注E.非常關注

2．藥品品牌（如：是否為知名品牌）：

A.根本不關注B.不太關注C.一般D.比較關注E.非常關注

3．藥品的產地：

A.根本不關注B.不太關注C.一般D.比較關注E.非常關注

4．藥品外觀（包裝）設計：

A.根本不關注B.不太關注C.一般D.比較關注E.非常關注

5．藥品是否進入《基本醫療保險藥品目錄》：

A.根本不關注B.不太關注C.一般D.比較關注E.非常關注

藥品屬性：

6．藥品的安全性：

A.根本不關注B.不太關注C.一般D.比較關注E.非常關注

7．藥品的療效：

A.根本不關注B.不太關注C.一般D.比較關注E.非常關注

8．藥品的劑型（服用方便性）：

A.根本不關注B.不太關注C.一般D.比較關注E.非常關注

9．藥品適應症：

A.根本不關注B.不太關注C.一般D.比較關注E.非常關注

10．藥品有效期長短：

A.根本不關注B.不太關注C.一般D.比較關注E.非常關注

11．藥品的副作用：

A.根本不關注B.不太關注C.一般D.比較關注E.非常關注

12．藥品的禁忌症：

A.根本不關注B.不太關注C.一般D.比較關注E.非常關注
