# Supplementary material for: Popularity and customer preferences for over-the-counter Chinese medicines perceived by community pharmacists in Shanghai and Guangzhou: a questionnaire survey study
Source: Chin Med. 2014 Sep 13;9:22. doi: 10.1186/1749-8546-9-22 (PMC4169131; doi:10.1186/1749-8546-9-22)
Supplement: Additional file 3 — Sales proportion in community pharmacies. [file 1749-8546-9-22-S3.docx]

**Supplementary file 3: Sales proportion in community pharmacies**

| Distribution items | Pharmaceutical sales / gross turnover | Prescription medicine sales / pharmaceutical sales | OTC medicine sales / pharmaceutical sales | OTC Chinese medicines sales / pharmaceutical sales |
| --- | --- | --- | --- | --- |
| 0-10% | 1% | 25% | 2% | 2% |
| 11-20% | 14% | 28% | 8% | 19% |
| 21-30% | 12% | 25% | 9% | 18% |
| 31-40% | 8% | 13% | 13% | 26% |
| 41-50% | 13% | 3% | 17% | 17% |
| 51-60% | 17% | 2% | 18% | 4% |
| 61-70% | 17% | 4% | 15% | 7% |
| 71-80% | 4% | 0 | 12% | 3% |
| 81-90% | 9% | 1% | 4% | 4% |
| 91-100% | 5% | 0 | 2% | 0 |

Source: Data analyzed from this survey study.
